# The Genetic Landscape of Fibrotic Interstitial Lung Diseases: Clinical Implications and Diagnostic Challenges in Familial Pulmonary Fibrosis

**DOI:** 10.3390/jcm15134951

**Published:** 2026-06-25

**Authors:** Claudio Tirelli, Ornella Rondinone, Fausta Alfano, Jacopo Cefalo, Giulia Nalesso, Matteo Ciracì, Carmine Salerni, Monica Rosa Miozzo, Stefano Centanni, Michele Mondoni

**Affiliations:** 1 Respiratory Unit, ASST Santi Paolo e Carlo, Department of Health Sciences, University of Milan, 20142 Milan, Italy; 2 Medical Genetics Unit, ASST Santi Paolo e Carlo, Department of Health Sciences, University of Milan, 20142 Milan, Italy

**Keywords:** familial pulmonary fibrosis, idiopathic pulmonary fibrosis, ILD, NGS, VUS, genetic counseling, precision medicine

## Abstract

The pathogenesis of interstitial lung diseases (ILDs) is significantly influenced by genetic factors, yet lack of consensus on the optimal timing for genetic testing and precise patient selection could hinder clinical practice. Position papers currently suggest testing patients presenting with a suspect of Familial Pulmonary Fibrosis (FPF) and with extra-pulmonary syndromic features (i.e., premature graying, cytopenias, liver cirrhosis) for genetic screening. Diagnostics rely on next-generation sequencing (NGS) to identify pathogenic/likely pathogenic variants in telomere-related and surfactant-related genes. A specialized genetic consultation is essential in the correct interpretation of test results, especially when variants of uncertain significance (VUS) are detected. Adoption of other tests, such as polygenic risk scores, could further support precision medicine in ILD care. Future research might address the knowledge gap regarding early test prescription and the role of therapy, including lung transplant stratification and antifibrotic therapy, in FPF.

## 1. Introduction

Interstitial lung diseases (ILDs) comprise a heterogeneous group of disorders, with different etiologies and characterized by varying degrees of inflammation and fibrosis. Typical manifestations of ILDs include progressive dyspnoea, cough, and impaired gas exchange, ultimately leading to restrictive lung volumes, chronic hypoxaemia, and respiratory failure [1,2].

Genetic factors are now recognized as risk factors in ILD pathogenesis, particularly in the context of familial aggregation. Moreover, Familial Pulmonary Fibrosis (FPF) is now recognized as a distinct clinical entity, defined by the presence of fibrotic ILD in at least two first- or second-degree relatives [2,3]. It is important to distinguish FPF from idiopathic pulmonary fibrosis (IPF): IPF refers to a specific chronic, progressive fibrosing interstitial pneumonia of unknown cause limited to the lungs and associated with a Usual Interstitial Pneumonia (UIP) pattern, whereas FPF is a clinical definition based on familial aggregation of any form of fibrotic ILD, regardless of the underlying radiological or histopathological pattern. These two entities can overlap, since IPF can present in a familial context, but they should be kept conceptually separate throughout the diagnostic process. However, in daily practice, a significant knowledge gap persists regarding the optimal timing for genetic screening and the precise selection of patients who would benefit from these investigations.

Over the last few decades, the study of ILD family clusters has led to the identification of numerous rare pathogenic variants and common polymorphisms linked to both familial and sporadic disease forms. Specifically, rare variants in telomere-related genes (TRGs) and surfactant-related genes (SRGs) are now established causes of progressive fibrosis [1,4]. In many cases, these genetic alterations manifest through specific extra-pulmonary signs, such as premature graying, cytopenias, or liver cirrhosis, which are highly suggestive of underlying telomere syndromes [5]. Furthermore, common polymorphisms, most notably in the MUC5B promoter, have emerged as major genetic risk factors not only in FPF but also for IPF [6]. These findings suggest that genomic variants may contribute to fibrosis through cellular homeostasis, genomic instability, and dysfunctional alveolar epithelial repair [7].

The integration of advanced genomic technologies, such as genome-wide association studies (GWAS) and next-generation sequencing (NGS), has accelerated the discovery of these risk factors and their associated molecular pathways. This synergy enables the development of precision medicine approaches aimed at stratifying patients based on their unique molecular profiles [6,7,8]. The integration of a specialized geneticist within the Multidisciplinary Team (MDT) is thus expected to soon become a key issue, not only for the interpretation of complex genomic data—such as variants of uncertain significance (VUS)—but also for navigating the profound ethical implications for the patient’s family.

The aim of this article was to review the current knowledge on the genetic factors underlying fibrotic ILDs, emphasizing the clinical utility of available genetic tests in detecting pathogenic variants and common polymorphisms. Furthermore, the critical role of genetic counseling and the emerging challenges linked to VUS detection have been explored, providing a comprehensive outlook on future therapeutic perspectives and patient management.

## 2. Methods

A non-systematic, narrative literature review was conducted. The search engines PubMed, Scopus and Web of Science were adopted to retrieve the most relevant, English-written, articles on these topics from international scientific journals. The search period covered publications from January 2000 to April 2026 (last access to search engines: 15 April 2026), with priority given to articles published within the last five years and to international guidelines and position papers. The search strategy included combinations of the following keywords and was limited to English-language articles: Interstitial Lung Disease; Pulmonary Fibrosis; Idiopathic Pulmonary Fibrosis; Familial Pulmonary Fibrosis; Genetic Pulmonary Fibrosis; Variant; Surfactant Related Genes; Telomerase Related Genes; Genetics; Next Generation Sequencing; Variant of Uncertain Significance; Telomeropathy. Studies were selected based on relevance to the genetic and epigenetic landscape of fibrotic ILD and FPF, with preference for original research, large cohort studies, systematic reviews, and consensus documents. Case reports were included only when they illustrated novel genotype–phenotype correlations. Additional eligible studies were retrieved from bibliographies of the most cited studies and book chapters. Two authors independently screened abstracts from all the collected articles (C.T.; O.R.); in the case of disagreement, a third senior author screened and decided whether to include it for the analysis. Full texts of the included articles were examined and relevant data summarized. As this is a narrative review, no formal quality assessment of individual studies was performed; references were chosen by author consensus to provide a clinically oriented synthesis. A potential selection bias related to the narrative nature of this synthesis cannot be excluded.

## 3. Genetic and Epigenetic Factors in the Pathogenesis of Fibrotic ILD

The pathogenetic framework of ILD, and more specifically fibrotic ILD, as idiopathic pulmonary fibrosis (IPF), has undergone a radical evolution, transitioning from a model of chronic inflammation to one centered on the failure of the alveolar epithelium. In approximately 10–20% of cases, pulmonary fibrosis (PF) manifests with a strong hereditary component, termed Familial Pulmonary Fibrosis (FPF). Aligned with the European Respiratory Society (ERS) statement, a 10-fold increase in disease prevalence could be observed among relatives of patients with pulmonary fibrosis, validating the significant burden of the familial component, as documented across major international cohorts [2]. However, even in sporadic cases, genetic variants act as critical modifiers that interact with environmental triggers—the so-called exposome—to dictate disease onset, clinical trajectory, and therapeutic response [9]. This complex genetic architecture is characterized by a continuum of risk, encompassing rare, highly penetrant pathogenic variants in causative genes primarily associated with FPF and common polymorphisms that act as significant risk alleles in the broader population.

### 3.1. The Role of Telomere Maintenance and Cellular Senescence

The primary pillar of PF pathogenesis, particularly in aging populations, is the dysfunction of telomere maintenance. Telomeres, the ribonucleoprotein structures at chromosomal ends, act as protective caps to prevent DNA degradation and fusion. In the context of the lung, the maintenance of telomere length is vital for the regenerative capacity of alveolar epithelial type 2 cells (AEC2s). Among telomere-related genes, the most frequent disease-causing variants are found in the *TERT* (Telomerase reverse transcriptase) and *TERC* (Telomerase RNA Component) genes, occurring in approximately 15% of FPF (with TERT clearly predominant over TERC) and in about 2% of sporadic IPF cases. Recent large cohort studies have refined the relative contribution of single TRGs: among genetically solved FPF cases, pathogenic variants are most frequently identified in *TERT* (~40–45%), followed by *RTEL1* (Regulator of Telomere Elongation Helicase 1) (~15%) and *PARN* (Poly(A)-specific ribonuclease) (~10–12%) and only secondarily in *TERC* (≤10%) and other genes such as *DKC1* (Dyskerin Pseudouridine Synthase 1), *TINF2* (TERF1-interacting nuclear factor 2) and *KIF15* (Kinesin Family Member 15) [3]. Pathogenic variants in *PARN* and *RTEL1*) are also recognized in fibrotic patients, since deficiencies in these proteins lead to a precipitous decline in telomere length.

**Figure 1:**
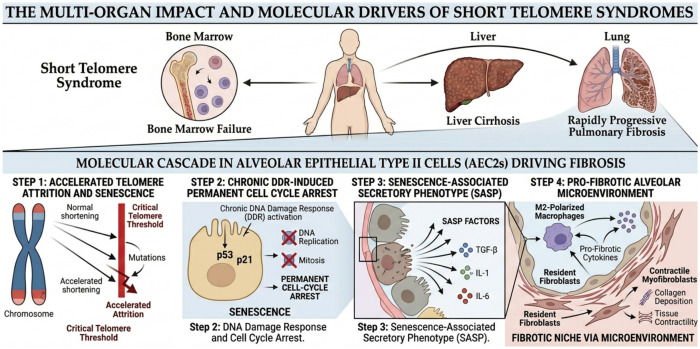
The systemic and molecular drivers of short telomere syndromes in pulmonary fibrosis. The precise molecular cascade within the alveolar microenvironment driving lung fibrosis is represented in a multi-step approach. Step 1: accelerated telomere attrition reaching a critical threshold. Step 2: chronic DDR activation leading to permanent cell-cycle arrest (senescence) mediated by the p53 and p21 (or p16INK4a) pathways. Step 3: development of the SASP and the secretion of pro-fibrotic factors (TGF-β, IL-1, IL-6). Step 4: establishment of a pro-fibrotic alveolar microenvironment, featuring the recruitment of M2-polarized macrophages and the activation of contractile myofibroblasts.

When pathogenic variants in these genes occur, clinically, the so-called short telomere syndrome can manifest. This often includes bone marrow failure, liver cirrhosis, and a rapidly progressive form of PF [1]. The molecular consequence of telomere attrition is the induction of AEC2 senescence. When telomeres reach a critical threshold, the DNA damage response (DDR) is chronically activated, leading to a permanent cell-cycle arrest mediated by the p53/p21 and p16INK4a pathways. These senescent AEC2s are not metabolically dormant; instead, they adopt a Senescence-Associated Secretory Phenotype (SASP). This phenotype is characterized by the profuse secretion of pro-inflammatory cytokines and pro-fibrotic growth factors, most notably Transforming Growth Factor Beta (TGF-β), Interleukin-1 (IL-1), and IL-6 [10]. This chronic secretome alters the alveolar microenvironment, promoting the recruitment of innate immune cells such as M2-polarized macrophages and the activation of resident fibroblasts into contractile myofibroblasts (Figure 1).

Furthermore, the phenomenon of genetic anticipation, whereby successive generations develop the disease earlier, is biologically plausible and frequently observed though potentially modulated by environmental and epigenetic influences [2].

### 3.2. The Impact of Surfactant Homeostasis Disruption

While telomere pathogenic variants typically drive adult-onset disease, disruptions in the surfactant system provide a critical link between pediatric and adult ILD, emphasizing the importance of epithelial proteostasis. Surfactant, a complex mixture of phospholipids and proteins (SP-A, SP-B, SP-C, SP-D), is essential for reducing alveolar surface tension. Pathogenic variants in *SFTPA1*, *SFTPA2*, *SFTPB*, *SFTPC* and the lipid transporter *ABCA3* disrupt the delicate balance of surfactant production and storage in lamellar bodies [11,12,13]. The majority of pathogenetic variants in *SFTPC* involve the BRICHOS domain, which is crucial for proper protein folding. When these proteins misfold, they accumulate within the lumen of the endoplasmic reticulum (ER), triggering a state of chronic ER stress and activating the Unfolded Protein Response (UPR) [12]. If the UPR fails to restore homeostasis, it triggers pro-apoptotic signaling and epithelial-to-mesenchymal transition (EMT). Interestingly, the inheritance of these variants shows high variability. *SFTPC* and *SFTPA* pathogenic variants usually follow an autosomal dominant pattern with incomplete penetrance. Hypomorphic *SFTPB* variants in the homozygous state can lead to adult-onset pulmonary fibrosis and should therefore be considered in the genetic differential diagnosis of FPF [13]. Moreover, pathogenic variants in *SFTPA1* and *SFTPA2* are significantly associated with the development of lung adenocarcinoma in smokers, highlighting how ER-stress-induced genomic instability can bridge the gap between fibrotic and neoplastic transformation [14].

### 3.3. Common Susceptibility Variants: The MUC5B and TOLLIP Axis

A landmark discovery in IPF genetics was the identification of common single-nucleotide polymorphisms (SNPs) that contribute to disease susceptibility in the general population. The most influential is the rs35705950 variant located in the promoter region of *MUC5B*, which encodes the major gel-forming mucin in the lung. This gain-of-function variant creates an enhancer region that dynamically binds the transcription factor FOXA2, leading to a 30- to 40-fold increase in MUC5B expression in the terminal bronchioles [15]. The excessive production of mucus impairs the mucociliary clearance mechanisms, thus causing the retention of inhaled particles, pollutants, and microbes in the distal airspaces. The reiteration of these processes triggers subclinical, repetitive alveolar injury [16].

Although the *MUC5B* T-allele is widely recognized as the strongest risk factor for developing IPF, its subsequent impact on clinical prognosis remains a subject of intense debate and controversy. Several cohorts described an apparent 2-to-3-fold survival advantage in *MUC5B* carriers; however, this survival paradox must be interpreted with extreme caution. Emerging evidence suggests that this observed benefit may be driven by lead-time bias, resulting from earlier diagnostic recognition due to subclinical disease awareness, or unmeasured clinical and environmental confounders rather than a true protective biological mechanism. This conflicting evidence represents a critical knowledge gap that prevents the usage of *MUC5B* status as a definitive prognostic biomarker in clinical practice, highlighting the need for future longitudinal functional studies [17].

In parallel, the Toll-interacting protein (*TOLLIP*) gene plays a crucial role in immune regulation. TOLLIP acts as an endogenous inhibitor of TLR2 and TLR4 signaling, thereby suppressing the overproduction of TNF-α and IL-6. Genetic variations in TOLLIP, as the rs5743890 minor allele, lead to reduced protein expression, correlating with reduced protection against reactive oxygen species (ROS) and mitochondrial damage in AEC2s [18]. Clinically, carriers of this TOLLIP variant exhibit more rapid FVC decline and a significantly higher mortality risk [19]. Furthermore, *TOLLIP* rs3750920 polymorphism has been recognized as a pharmacogenetic biomarker, able to influence the efficacy of antioxidants like N-acetylcysteine [18].

### 3.4. Epigenetic Regulation in ILD Pathogenesis

Epigenetic modifications, defined as heritable changes in gene expression that do not alter the DNA sequence, have been proposed as a potential interface between environmental exposures and the fibrotic genome. Three main epigenetic mechanisms have been associated with the persistent activation of profibrotic myofibroblasts in ILD: DNA methylation, histone modifications, chromatin remodeling, and non-coding RNA regulation. It should be noted that most of the available evidence is associative and derived from preclinical or cross-sectional human studies; a direct causal role of these epigenetic changes in driving fibrosis in humans remains to be definitively established.

DNA methylation has been recognized as significantly altered in IPF. Hypermethylation of tumor suppressor genes and hypomethylation of profibrotic genes stimulate uncontrolled matrix deposition. Notably, methylation changes in the *MUC5B* promoter correlate with its overexpression [19].

Histone acetylation and methylation regulate DNA expression. In fibrotic lungs, the overexpression of Histone Deacetylases (HDACs) leads to the silencing of antifibrotic genes, such as *PTGER4* (the receptor for Prostaglandin E2), which normally inhibits fibroblast activation. This epigenetic silencing makes the lung tissue resistant to endogenous anti-inflammatory signals, perpetuating the fibrotic cycle [20].

In ILD, the downregulation of the microRNA miR-29 family (which targets multiple collagen genes) and the miR-200 family (which maintains epithelial identity) allows for the unopposed induction of epithelial–mesenchymal transition (EMT) [21]. Furthermore, long non-coding RNAs (lncRNAs) are emerging as key regulators of the DNA damage response in senescent AECIIs, bridging the gap between telomere dysfunction and epigenetic reprogramming [22].

## 4. Clinical Features in Familial Pulmonary Fibrosis

While adults with FPF often present with clinical, radiological, and histopathological features indistinguishable from sporadic cases, the familial form is frequently characterized by an earlier age of onset and a more aggressive, progressive phenotype with a poorer prognosis. Crucially, FPF patients, particularly those harboring telomere-related gene (TRG) variants, often exhibit a spectrum of extrapulmonary manifestations, reflecting the multisystemic nature of these genetic disorders (Table 1) [23,24,25,26].

### 4.1. Clinical Features in Carriers of Telomere-Related Genes Variants

TRG pathogenic variants represent the most prevalent genetic basis for FPF, accounting for approximately 25–30% of cases [2]. Due to this prevalence, their clinical spectrum is well-characterized, often falling under the umbrella of “short telomere syndromes.”

Hematologic involvement is the most significant extrapulmonary manifestation. Patients frequently present with anemia (17–27%), macrocytosis (24–41%), and thrombocytopenia (8–54%) [27,28,29]. Hematologic abnormalities appear more frequent in carriers of *DKC1*, *TINF2*, and *TERC* pathogenic variants compared to *TERT*, *PARN*, or *RTEL1* [28]. Furthermore, these patients face a higher risk of hematologic malignancies, specifically myelodysplastic syndromes and acute leukemia [27,30].

Liver involvement ranges from asymptomatic enzyme elevation (5–27%) to cirrhosis and hepatopulmonary syndrome [28,31]. Additionally, premature hair greying (typically before age 30) occurs in 15–40% of carriers and should serve as a key red flag during clinical history taking [32].

Beyond the classic triad, short telomere syndromes may encompass primary immunodeficiency, retinal disorders, and neurological impairment [33].

### 4.2. Clinical Features in Carriers of Surfactant-Related Gene (SRG) Variants

Pathogenic variants in genes regulating surfactant homeostasis (*ABCA3*, *NKX2-1*, *SFTPA1/SFTPA2*, *SFTPB* and *SFTPC*) are strongly linked to ILD, often associated with an increased susceptibility to lung cancer [14,34,35,36,37,38]. While *SFTPA1* and *SFTPA2* pathogenic variants typically manifest in adulthood, *SFTPC* pathogenic variants cause approximately 1–5% of ILD cases both in pediatric and adult patients [35,36,37,38,39,40].

Biallelic pathogenic variants of *ABCA3* are most commonly associated with fatal neonatal respiratory distress; however, the clinical severity is variable and can also present as ILD in children and adults, typically presenting with ground-glass opacities, septal thickening, and parenchymal cysts on chest computed tomography (CT) [41,42,43].

Finally, pathogenic variants in *NKX2-1* transcription factor are the hallmark of the “brain–lung–thyroid syndrome”, which is characterized by choreoathetosis, hypothyroidism and ILD. Notably, ILD may be the isolated clinical manifestation in 25% of cases [44,45,46].

Recently, *TBX4* pathogenic variants have emerged as a cause of pulmonary fibrosis, frequently associated with pulmonary hypertension and distinctive central bronchiectasis [47,48]. Although *SFTPB* has classically been associated with severe neonatal disease, hypomorphic *SFTPB* variants in the homozygous state can lead to adult-onset pulmonary fibrosis [49]. Of note, *SFTPC*-related disease can present in both pediatric and adult patients within the same kindred, with a phenomenon resembling clinical anticipation analogous to that observed in TRG carriers; the underlying biological basis remains unclear and likely reflects a combination of modifying genetic and environmental factors [3,35].

### 4.3. Convergent Pathways: Fibrosis and Lung Cancer

The epidemiological link between FPF and lung cancer is underpinned by shared molecular pathogenetic hallmarks. Genomic instability, driven by telomere attrition, can promote the clonal expansion of cells with malignant potential [50]. Pathogenic variants in *SFTPA1* and *SFTPA2* significantly impact on the development of lung adenocarcinoma in smokers, mainly through ER-stress-induced genomic instability. Furthermore, *TP53* and members of the JAK-STAT pathway are frequently mutated or dysregulated in both conditions. During fibrogenesis, NKX2-1 (HGNC-approved gene symbol; also historically referred to as TTF-1, Thyroid Transcription Factor-1, at the protein level) is also overexpressed, and this might alter the regulation of surfactant protein production [51]. Moreover, Matrix Metalloproteinase 1 (MMP1), crucial for the invasive potential of cancer cells, is highly expressed in the early stages of IPF, facilitating epithelial-to-mesenchymal transition and angiogenesis [52].

Finally, the epigenetic mechanisms which are active in ILD have been also linked to lung cancer development. Global methylation studies have confirmed this overlap, identifying hundreds of CpG islands that are identically methylated in both FPF and adenocarcinoma [53]. Of notice, tyrosine kinase inhibitors like nintedanib are used as effective antifibrotic, though being originally developed for oncology [54].

## 5. Radiologic Features in Familial Pulmonary Fibrosis

The radiological landscape of FPF is heterogeneous, with patterns that often overlap but sometimes offer specific clues to the underlying genetic driver. In clinical practice, high-resolution computed tomography (HRCT) patterns are classified according to international guidelines to reflect specific morphological distributions. A Usual Interstitial Pneumonia (UIP) pattern is strictly defined by basal- and peripheral-predominant reticulation, traction bronchiectasis, and honeycombing, while a probable UIP pattern lacks honeycombing but shares the same distribution. Cases with features suggestive of fibrosis that do not meet these criteria or present atypical features are classified as indeterminate for UIP. Conversely, a Nonspecific Interstitial Pneumonia (NSIP) pattern typically exhibits bilateral, symmetric ground-glass opacities (GGOs), fine reticulation, and characteristic subpleural sparing [55]. The UIP pattern is common to several forms of ILD, both idiopathic and secondary to connective tissue diseases and vasculitis [56,57]. Of notice, the UIP pattern remains also the most frequent radiological manifestation associated with TRG pathogenic variants, occurring in approximately 46–55% of carriers [58]. However, TRG-related disease is notable for its pleiomorphic presentation; atypical features are reported in up to 20% of cases and include peribronchovascular distribution of fibrosis, lower-lobe predominance, and features of pleuroparenchymal fibroelastosis (PPFE) [58].

Furthermore, the Combined Pulmonary Fibrosis and Emphysema (CPFE) phenotype is reported in approximately 14–40% of TRG variant carriers who are smokers, with male preponderance, which is higher than the prevalence of CPFE observed in unselected TRG cohorts and in smokers without TRG variants. The relative contribution of TRG variants, sex and smoking to this phenotype has not yet been disentangled in large prospective cohorts [1,2]. TRG pathogenic variants have also been documented in patients with NSIP, Hypersensitivity Pneumonitis (HP), or indeterminate patterns [59].

In contrast, ILD associated with SRG pathogenic variants presents a more varied radiological spectrum, highly influenced by the patient’s age at onset. In pediatric and childhood ILD secondary to *ABCA3*-related or *TBX4*-related pathogenic variants, a radiological NSIP pattern (or ground-glass predominance) is observed significantly more often than a UIP pattern. Adult carriers of *SFTPC* and *ABCA3* variants frequently display an indeterminate pattern with a combination of diffuse ground-glass attenuation, interlobular septal thickening, and variably sized, thin-walled parenchymal cysts [60,61]. These cysts are often subpleural or embedded within areas of GGO, predominantly in the lower lobes [61]. Finally, specific developmental or rare variants may present with distinctive signs, such as the central bronchiectasis and concomitant pulmonary hypertensive vascular changes frequently associated with *TBX4* pathogenic variants [48,62]. The main radiologic findings according to specific gene pathogenic variants are summarized in Table 1.

## 6. Current Indications for Genetic Testing in Familial Pulmonary Fibrosis

Currently, the framework for genetic evaluation is primarily defined by the European Respiratory Society (ERS) Statement on Familial Pulmonary Fibrosis (2023) [2] and the Pulmonary Fibrosis Foundation (PFF) Position Paper (2022) [60]. These documents aimed to standardize the heterogeneous approaches previously used in clinical practice. In particular, the ERS statement identifies four key clinical domains that should trigger the consideration for genetic sequencing in patients with ILD [61]. When a patient falls into one or more of these categories, the diagnostic yield for identifying a pathogenic variant is significantly higher:

(1) Family History (Familial ILD): Patients with fibrotic ILD who have at least one first- or second-degree relative with any form of fibrotic ILD;
(2) Early Onset of Disease: Individuals presenting with IPF or other fibrotic ILDs at a young age (typically defined as <50 years), even in the absence of a documented family history;
(3) Extrapulmonary Manifestations (Telomeropathy Spectrum): Patients with ILD (sporadic or familial) who exhibit personal or family histories of features associated with short telomere syndromes. These include bone marrow failure, unexplained cytopenias, macrocytosis, liver cirrhosis, or premature hair greying (typically <30 years);
(4) Known Familial Pathogenic Variants: when a (likely) pathogenic variant has already been identified in a proband, testing of relatives should be performed as predictive (presymptomatic) testing, typically as targeted analysis of the known familial variant. In this setting the diagnostic yield is naturally very high because only one variant is being interrogated; this approach is distinct from diagnostic sequencing in symptomatic individuals and is permissible in asymptomatic adult relatives only after appropriate genetic counseling.

Additionally, testing is recommended in cases where pulmonary fibrosis is associated with lung adenocarcinoma, particularly when there is a family cluster. No consensus exists on routine testing for common variants (e.g., MUC5B) or evaluation before lung transplantation [60].

A critical development concerns telomere length (TL) testing. Multiple studies have demonstrated a significant pharmacogenomic interaction: patients with short age-adjusted TL who receive immunosuppressants (such as mycophenolate mofetil) face increased mortality [62,63,64]. Furthermore, TL can be considered a biomarker to predict aggressive disease progression and post-transplant complications, such as chronic lung allograft dysfunction [65,66]. However, no consensus has been reached as to whether TL testing should become routine in the pre-transplant evaluation and for all f-ILD patients.

Despite its clinical relevance, the widespread implementation of TL testing is severely limited by significant inter-platform variability and a profound lack of global standardization [2]. Currently, methodologies vary from high-throughput but relatively variable quantitative PCR (qPCR) assays to more precise, cell-fraction-specific Flow-FISH, leading to potential inter-laboratory discrepancies [2]. Because reference curves for healthy populations are often center-specific, establishing universal clinical utility thresholds remains challenging. In clinical practice, while a telomere length below the 10th percentile serves as a sensitive screening threshold to raise clinical suspicion of an underlying genetic etiology, a drop below the 1st percentile represents the critical clinical pivot. This strict cut-off is strongly associated with full-blown syndromic telomeropathies, rapid disease progression, and an exceptionally high risk of severe bone marrow failure or immunosuppressive toxicity following lung transplantation [64]. Moreover, no universally accepted age-adjusted cut-offs are available, and reference distributions are largely derived from populations of European ancestry [2]. These constraints currently limit the cross-center comparability of results and highlight the need for international standardization before TL can be routinely integrated into clinical decision-making.

Genetic sequencing should not be an isolated laboratory request, but it might be integrated into a Multidisciplinary Team (MDT) discussion involving pulmonologists, radiologists, pathologists and, for selected cases, geneticists. This collaborative approach is essential for the correct interpretation of variants and for managing the complex clinical and psychological implications for the patient’s family [56,67,68,69].

The decision to perform genetic testing should be balanced between diagnostic yield and the psychological impact on the patient, in view of the pre-test probability. Recent cohort studies suggest that even a single relative with a history of pulmonary disease might raise suspicion. In FPF, the pattern of inheritance is typically autosomal dominant with incomplete penetrance, meaning the disease may skip generations or present with different degrees of severity [2]. Assessing a three-generation pedigree is today considered by geneticists the most cost-effective diagnostic tool.

While <50 years is the standard threshold, testing in sporadic cases between 50 and 60 years is increasing, since approximately 10% of apparently sporadic IPF patients in this age might carry pathogenic TRG variants, especially in the presence of extrapulmonary comorbidities [61].

Genetic testing is today adopted also in the pre-transplant workup, since TRG pathogenic variants enhance the risk for post-transplant marrow failure and chronic lung allograft dysfunction (CLAD) due to impaired lymphocyte proliferative capacity [66].

## 7. Molecular Methods for Genetic Testing in Familial Pulmonary Fibrosis

Modern diagnostic strategies are increasingly adopting three main NGS-based levels of resolution: targeted gene panels, exome sequencing and genome sequencing (Table 2).

**Figure 2:**
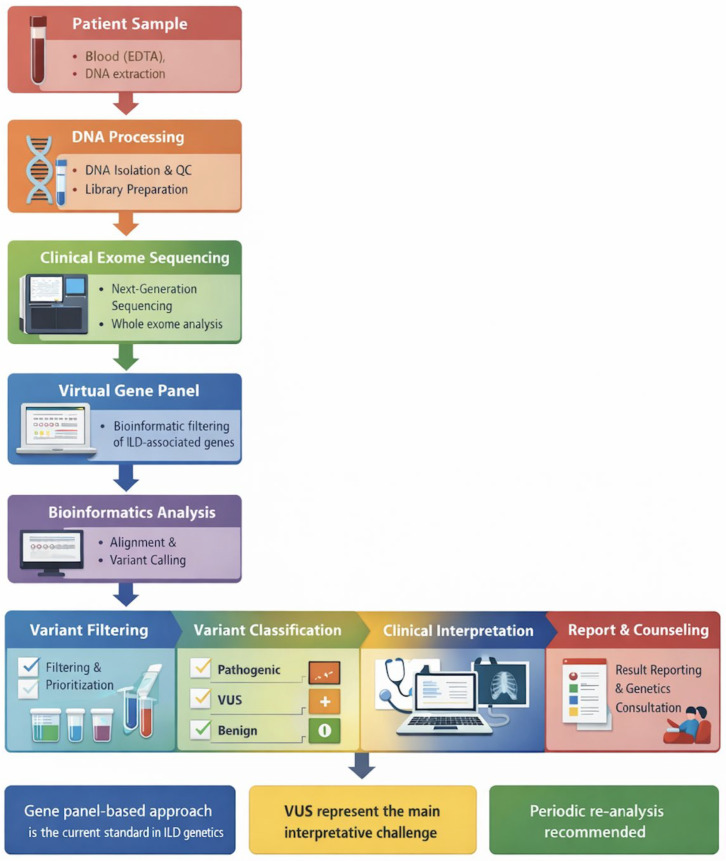
The next-generation sequencing diagnostic workflow for genetic testing in interstitial lung disease using clinical exome sequencing. The flowchart illustrates the sequential steps from patient sampling to clinical reporting. Patient sample and DNA processing: The process begins with blood collection followed by DNA extraction, quality control (QC), and library preparation. After that, clinical exome sequencing (CES) is performed via high-throughput next-generation sequencing (NGS) to analyze the whole exome. A virtual gene panel approach is applied to filter sequencing data for specific ILD-associated genes. This is followed by alignment to a reference genome and variant calling. Identified variants undergo a multi-step evaluation involving filtering, prioritization, and classification into pathogenic, VUS (Variant of Uncertain Significance), or benign categories according to established guidelines. Findings are then integrated with clinical and radiological data for final interpretation. The final results are delivered through a formal genetic report and clinical consultation.

Targeted gene panels (TGPs) focus on a specific set of genes known to be associated with ILD (Figure 2). Commonly, these panels include up to 30 genes; among these, the most are described as associated to PF (i.e., TRGs and SRGs). Typical core genes covered by FPF/IPF multi-gene panels include the telomere-related genes *TERT*, *TERC*, *RTEL1*, *PARN*, *DKC1*, *TINF2*, *ACD*, *NAF1*, *NHP2*, *NOP10*, and *ZCCHC8* and the surfactant- and developmental-related genes *SFTPA1*, *SFTPA2*, *SFTPB*, *SFTPC*, *ABCA3*, *NKX2-1* and *TBX4*. Importantly, modern TGPs frequently incorporate relevant regulatory regions, especially when they are established hotspots for pathogenic or susceptibility variants, such as the *MUC5B* promoter. While restrictive, these panels offer a very high depth of coverage (i.e., the number of times a specific nucleotide is sequenced), ensuring high sensitivity for detecting pathogenic variants even in complex regions. However, they cannot identify new candidate genes or variants outside the pre-selected list [69].

Exome sequencing (ES) covers the exome, which includes all protein-coding regions of the genome (approximately 1–2% of the total DNA). Historically, ES data have been filtered through in silico virtual panels to focus on known ILD genes first while allowing the raw data to be re-analyzed later as new genes are discovered [70]. However, if all relevant causative ILD genes are already characterized, ES may not offer a clear clinical advantage over a well-designed targeted gene panel, which typically guarantees superior coverage depth and higher analytical sensitivity. Therefore, ES is today best prioritized for research purposes, including atypical or unsolved clinical cases and novel gene discovery.

Genome sequencing (GS) thus covers both coding (exons) and non-coding (introns and regulatory regions) DNA. GS is also able to capture non-coding regulatory variants, such as those in the promoter regions of *TERT* or *MUC5B*, as well as structural variations (SVs), including large deletions or duplications, which are often missed by targeted panels or ES. Furthermore, GS provides reliable coverage of non-coding genes such as *TERC*, which is frequently underrepresented in ES, and some GS pipelines can directly estimate telomere length from sequencing reads, providing additional supportive information for variant interpretation in suspected telomeropathies [71].

Finally, long-read sequencing (LRS) technologies (e.g., Oxford Nanopore, PacBio) are emerging. Beyond its ability to span complex, G-rich repetitive regions within the *TERT* promoter or distal telomeric sequences, the advantages of LRS extend to the superior detection of large structural variants (SVs) and the unique ability to resolve the exact genomic location of segmental duplications—chromosomal complexities that standard short-read genome sequencing (GS) often struggles to map accurately [72]. This technological leap is essential for a complete characterization of the telomeropathy spectrum, where the length and stability of repetitive sequences are directly linked to the fibrotic phenotype.

### 7.1. Variant Classification According to the ACMG Criteria

To ensure clinical consistency, genetic variants in ILD are categorized according to the framework established by the American College of Medical Genetics and Genomics (ACMG). This system utilizes a five-tier classification based on multiple lines of evidence, including population frequency, computational predictions, functional studies, and family segregation data (Table 3) [73]:

Class I (benign) and Class II (likely benign) variants are frequently found in the general population or have been proven to have no impact on protein function. They are not reported as causative agents for pulmonary fibrosis.

Class III (Variant of Uncertain Significance—VUS) variants represent a diagnostic grey zone, as VUSs are variants where the evidence on clinical impact and pathogenicity is either insufficient or conflicting. With the actual knowledge, the detection of a VUS represents a notable clinical challenge due to insufficient or conflicting evidence. In clinical practice, it is useful to differentiate VUS into those with true conflicting uncertainty and those with supportive but insufficient evidence (e.g., rare variants with phenotype or telomere length match). While the latter may help guide a working diagnosis and family segregation testing in symptomatic individuals, they must never be used for predictive testing in asymptomatic relatives, especially children (see Table 3 for a detailed clinical management schema).

Class IV (likely pathogenic) and Class V (pathogenic) variants have a high probability (>90% for Class IV) or near-certainty (Class V) of being disease-causing. Only these variants should be utilized for clinical decision-making or performing predictive testing in relatives.

### 7.2. Interpretation of Genetic Test Results and the Approach to Complex Genomic Data

The clinical utility of NGS is frequently challenged by the high prevalence of VUS, which in ILD cohorts can reach or exceed 30%, creating significant management dilemmas for clinicians and anxiety for patients [74,75,76,77,78]. The “grey zone” status of a VUS means it cannot be used to inform cascade testing in asymptomatic relatives, as its presence does not guarantee the development of disease, nor does its absence rule out risk. To resolve these interpretation gaps, a modern genotype–phenotype integration is required. Beyond traditional segregation analysis, several advanced strategies are currently employed:

Biomarker Correlation: In cases of a VUS in a TRG, measuring lymphocyte telomere length (LTL) via flow-FISH is a critical tie-breaker. As established in clinical workflows, a variant associated with an LTL dropping below the 1st–10th percentiles provides compelling biological cross-evidence of structural dysfunction, potentially supporting the clinical actionability of the variant even when formal ACMG criteria provisionally remain at Class III [79].

Polygenic Risk Scores (PRS) and Common Susceptibility Loci: The genetic architecture of ILD is increasingly recognized as a complex continuum. Beyond rare high-penetrance monogenic drivers, the cumulative impact of polymorphisms in common susceptibility loci—most notably the *MUC5B* promoter variant (rs35705950)—is today quantified through polygenic risk scores (PRSs). These scores aggregate the risk of thousands of common, low-effect variants to help explain the incomplete penetrance observed within families and predict disease progression. However, PRS is currently limited by a lack of international standardization and a profound European ancestry bias, meaning it is not yet ready for routine diagnostic application in asymptomatic relatives [78].

AI-Driven Proteogenomics: Traditional computational predictors (i.e., PolyPhen) are being replaced by deep-learning tools, which provide a higher level of confidence in predicting functional loss [74].

Dynamic Reclassification: Variant interpretation should require periodic re-evaluation, since databases such as ClinVar and ClinGen are constantly updated as new data emerge. Genetic reports should thus be re-evaluated every 12–24 months [75].

Functional Assays: Although largely confined to research settings, functional studies (i.e., measuring telomerase activity in vitro or surfactant protein secretion in cell models) remain the final way to prove a variant’s impact.

The detection of a VUS should therefore be viewed as a starting point to be evaluated in specialized multidisciplinary discussion.

## 8. Genetic Counseling and Clinical Management Post-Testing

The disclosure of genetic results in ILD is a high-stakes clinical encounter that requires a specialized multidisciplinary approach. Once a pathogenic (Class V) or likely pathogenic (Class IV) variant is identified, the focus shifts from diagnosis to long-term risk management and family health [76].

Modern multi-gene NGS panels applied to ILD cohorts frequently identify a significant proportion of VUS, with frequencies fluctuating between 15% and 35% depending on panel size [2]. Resolving this clinical ambiguity requires a rigorous, case-by-case application of the standard ACMG/AMP interpretation frameworks [76].

In patients with TRG pathogenic variants, clinical vigilance must be extended beyond the lungs, looking for other “short telomere” complications, including complete blood counts with differential to monitor for bone marrow failure and liver function tests/elastography to detect cirrhosis [2]. Furthermore, the presence of TRG variants may influence transplant decisions, as these patients are at higher risk for post-transplant cytopenias and immunosuppression-related toxicities [77]. In case SRG pathogenic variants are suspected, the management must focus also on lung cancer surveillance [14].

The detection of VUS requires a multidisciplinary discussion (with at least pulmonologist, radiologist, clinical geneticist, and molecular biologist) [67].

A crucial aspect of post-test counseling is the screening of first-degree relatives. Since familial ILDs often follow an autosomal dominant pattern with incomplete penetrance and variable expressivity, the presence of a pathogenic variant does not guarantee disease development. Counseling must carefully explain that a positive result in an asymptomatic relative indicates “at-risk” status, necessitating longitudinal follow-up, which also includes pulmonary function tests in asymptomatic first-degree relatives and HRCT in symptomatic ones to detect early ILA [78]. A negative result in the context of a known familial pathogenic variant provides definitive reassurance, removing the need for further clinical surveillance [79].

The psychological burden of pre-symptomatic diagnosis should be carefully considered. Relatives often experience significant anxiety regarding their future health. Furthermore, the discovery of a germline pathogenic variant may raise difficult questions regarding reproductive choices, including the possibility of prenatal or preimplantation genetic testing in selected familial settings. Additional issues that deserve explicit discussion before and after testing include the potential implications for insurance and employment (which vary by jurisdiction and may pose discrimination concerns), the disclosure of incidental or secondary findings (e.g., cancer predisposition variants identified during ES/GS), the practical limitations of predictive testing in asymptomatic carriers given incomplete penetrance and variable expressivity, and the complexity of managing variants of uncertain significance over time. The geneticist should ensure that the patient and their family are not only informed of the test results but also supported through the ethical complexities of genomic testing [80].

## 9. Future Perspectives

Future advancements in the field of genetics in ILD are expected to be focused on integrating multiomic data into clinical decision-making towards a mechanism-based precision medicine.

Advances in molecular profiling are reshaping diagnostic workflows. Gene-expression-based classifiers, particularly those derived from transbronchial biopsies, have demonstrated high accuracy in identifying Usual Interstitial Pneumonia (UIP), providing a minimally invasive complement to traditional imaging and histopathology [81,82]. Beyond diagnosis, the implementation of peripheral blood telomere length measurement and polygenic risk scores (PRSs) offers a robust framework for identifying individuals at high risk for rapid disease progression or the early development of interstitial lung abnormalities (ILAs) [83,84,85].

The success of targeted therapies in other respiratory conditions, such as cystic fibrosis, alpha 1 antitrypsin deficiency and acid sphingomyelinase deficiency, paves the way for substitutive and gene-directed approaches in ILD [86,87,88,89]. No specific data are available on the therapeutic response of FPF patients to standard therapies during acute exacerbations [90,91]. Potential strategies currently under preclinical investigation include gene silencing approaches targeting *MUC5B* and small molecules tailored to specific dysregulated pathways. It is important to underline that none of these strategies have yet reached clinical validation in FPF or IPF, and any extrapolation to clinical practice would currently be speculative [92].

### Ancestry Bias and Generalizability Challenges

A critical limitation of the current evidence base is that to date, the vast majority of large-scale genome-wide association studies (GWAS) and clinical cohorts in FPF, as well as *MUC5B* and polygenic risk score studies, have been restricted to populations of European descent. This lack of diversity severely impairs the clinical generalizability of genomic discoveries, affects the interpretation of allele frequencies and variant pathogenicity in non-European populations, and limits the generalizability of current diagnostic and predictive tools. Thus, since linkage disequilibrium patterns and allele frequencies vary significantly across diverse ethnicities, a risk score optimized for European cohorts cannot be reliably used to guide clinical decisions in patients of African, Asian, or Hispanic descent. In this sense, the integration of long-read sequencing technologies will be helpful for building comprehensive, ancestry-agnostic reference genomes, which represents a mandatory step toward achieving equitable personalized medicine in ILDs. Moreover, the adoption of adequately trained AI-based models in the interpretation of VUS might help in the struggle to differentiate benign population-specific polymorphisms from true pathogenic variants in non-European individuals.

In conclusion, increasing the representation of non-European ancestries in genomic studies and expanding the ethical debate on the complexity of testing patients is a priority to ensure equitable access to precision medicine [93].

## 10. Methodological Gaps and Current Challenges in ILD Evidence

As the field of ILD genomics moves toward clinical translation, several critical evidence gaps and methodological limitations must be addressed. First, much of the currently available data relies heavily on large, heterogeneous observational cohorts. While these cohorts helped in the identification of key genetic risk factors (i.e., *MUC5B* and telomere-related variants), they often lack standardized phenotyping, leading to potential confounding factors regarding disease progression and treatment responses across different centers. Furthermore, a significant gap is still present between observational association data and robust mechanistic evidence; how specific rare variants dynamically alter the alveolar microenvironment in humans remains partially speculative. Finally, there is a striking lack of prospective clinical trials evaluating genetics in ILD, particularly concerning the actual therapeutic response of FPF patients to standard antifibrotic therapies during acute exacerbations [90,91]. Taken together, all these factors inevitably affect the overall evidence grading when developing a comprehensive review of the current literature. Transitioning from purely descriptive clinical correlations to standardized, multi-center validated evidence will be crucial to establish definitive guidelines for routine genetic counseling and personalized patient management.

## 11. Conclusions

In conclusion, the integration of genetic testing into the diagnostic workup of fibrotic ILDs is emerging as a powerful instrument to better identify a subset of disease which is characterized by pulmonary and extra-pulmonary manifestations in FPF family members. Multicenter studies on the epidemiology of FPF together with expanding knowledge on the potentiality of these molecular tools are required. Standardizing genetic testing and establishing international consensus guidelines will be paramount to transforming these technological advances into improved longitudinal care and personalized therapeutic strategies.

## Figures and Tables

**Table 1:** Clinical impact, extrapulmonary involvement and radiologic patterns in Familial Pulmonary Fibrosis. AD, autosomic dominant; AR, autosomic recessive; UIP, Usual Interstitial Pneumonia, NSIP, Nonspecific Interstitial Pneumonia; PPFE, Pleuro-parenchymal Fibroelastosis; CPFE, Combined Pulmonary Fibrosis and Emphysema.

Gene Family	Genes	Inheritance	Typical HRCT Pattern	Extrapulmonary Involvements	Clinical Impact/Cancer Risk
Telomere-Related	*TERT*, *TERC*, *RTEL1*, *PARN*, *DKC1*, *TINF2*	AD/AR/X-linked	UIP, NSIP, PPFE, CPFE	Premature graying, macrocytosis, thrombocytopenia, liver cirrhosis	High risk of post-transplant complications and BMF
Surfactant-Related	*SFTPC*, *SFTPB*	AD/AR	Indeterminate UIP, NSIP	None (lung-specific)	SFTPC associated with adult fibrosis
	*SFTPA1*, *SFTPA2*	AD	UIP, NSIP	None (lung-specific)	Very high risk of lung adenocarcinoma
	*ABCA3*	AR	Predominant NSIP, thin-walled parenchymal cysts (often subpleural).	None (lung-specific)	High risk of pediatric ILD
Transcription Factors	*NKX2-1*	AD	cysts, NSIP	Hypothyroidism, choreoathetosis	“Brain–lung––thyroid” syndrome
Developmental Genes	*TBX4*	AD	NSIP	Small patella, skeletal anomalies	Association with pulmonary hypertension
Mucin Production	*MUC5B* (rs35705950)	Common polymorphism	UIP	None	Strongest risk factor for sporadic IPF; better prognosis

**Table 2:** Next-generation sequencing (NGS)-based genetic diagnostic strategies available in Familial Pulmonary Fibrosis: targeted gene panels (TGPs), exome sequencing (ES) and genome sequencing (GS). TRGs: telomerase-related genes; SRGs: surfactant-related genes; ILD: interstitial lung disease; TERT: telomerase reverse transcriptase; LRS: long-read sequencing; FPF: Familial Pulmonary Fibrosis.

Feature	Targeted Gene Panel (TGP)	Exome Sequencing (ES)	Genome Sequencing (GS)
Genomic region analyzed	Specific ILD-associated genes (~30 genes)	All protein-coding regions (~1–2% genome)	Entire genome (coding + non-coding)
Primary goal	Detect known pathogenic variants in selected genes	Identify coding variants + novel genes	Comprehensive analysis of all variants
Typical targets	TRGs and SRGs	~20,000 coding genes	All genomic elements including regulatory regions
Sequencing depth	Very high	Moderate	Lower (whole-genome coverage)
Sensitivity for known pathogenic variants	Very high	High	High
New gene discovery	No (limited to panel)	Yes (coding regions)	Yes (coding + non-coding)
Regulatory variants (TERT, MUC5B)	No	Limited	Yes
Structural variants (SVs)	Limited	Partial detection	Best detection
Repeat regions (telomeres, TERT promoter)	Limited	Challenging	Improved (especially with LRS)
Reanalysis potential	Limited	Yes	Yes
Diagnostic yield (ILD/FPF)	~10–20%	~15–30%	Potentially higher
Advantages	Fast, cost-effective, high sensitivity	Balance cost vs. discovery	Most comprehensive
Limitations	No novel genes, limited scope	Misses non-coding regions	High cost, complex analysis; lower sequencing depth makes detection of low-level mosaicism unlikely
Comparative costs	Low	Medium	High
Turnaround time	Fast (1–2 weeks)	Moderate (2–4 weeks)	Longer (4–8+ weeks)
Clinical use	First-line in suspected familial ILD	Extended diagnostic testing	Complex/unresolved cases
Best suited for	Known phenotype	Research (atypical or unsolved cases; novel gene discovery)	Research, telomeropathies, detection of structural variants, resolving genomic locations of duplications, and spanning G-rich repetitive regions (e.g., TERT promoter

**Table 3:** Genetic variants classification according to the framework established by the American College of Medical Genetics and Genomics (ACMG). VUS: Variant of Uncertain Significance; BA1 (stand-alone benign criterion): Allele frequency is >5% in a large, general population database (e.g., gnomAD). BS1–BS4 (strong benign criteria): BS1: Allele frequency is greater than expected for the disease; BS2: Observed in a healthy adult individual; BS3: Well-established in vitro or in vivo functional studies show no damaging effect; BS4: Lack of segregation in affected family members. BP1–BP7 (supporting benign criteria): BP1: Missense variant in a gene for which primarily truncating variants are known to cause disease; BP2: Observed in trans with a pathogenic variant for a fully penetrant dominant disease or in cis with a pathogenic variant; BP3: In-frame deletions/insertions in a repetitive region without known function; BP4: Multiple lines of computational (in silico) evidence show no deleterious effect; BP5: Variant found in a case with an alternate molecular cause; BP6: Reputable source as benign without independent evaluation; BP7: Synonymous variant with no predicted splice-site impact. PM2 (moderate pathogenic criterion): Absent or extremely rare in large, general population control databases. PP1–PP4 (supporting pathogenic criteria): PP1: Co-segregation with disease in multiple affected family members; PP3: Multiple lines of computational (in silico) evidence predict a deleterious effect on the protein; PP4: Patient’s phenotype or specific clinical markers (e.g., critically short telomere length) are highly specific for a disease with a single genetic cause. PS1–PS4 (strong pathogenic criteria): PS1: Same amino acid change as a previously established pathogenic variant; PS2: De novo variant (with confirmed paternity/maternity); PS3: Well-established in vitro or in vivo functional studies demonstrate a damaging effect on protein function; PS4: The prevalence of the variant in affected individuals is significantly increased compared to controls.

Class	Definition	Evidence Criteria	Clinical Interpretation	Use in Clinical Practice
Class I	Benign	Allele frequency above expected (BA1, BS1); no functional impact (BS3)	Not disease-causing	Not reported.
Class II	Likely Benign	Evidence against pathogenicity (BS1–BS4, BP1–BP7)	Unlikely disease-causing	Not used for diagnosis.
Class III (VUS)	Variant of Uncertain Significance with conflicting evidence	Conflicting evidence (combinations of benign and pathogenic criteria)	True uncertainty	Not for diagnosis or screening.
Class III (VUS)	Variant of Uncertain Significance with supportive but insufficient evidence	Insufficient evidence supported by combinations of PM2 (rare) *, PP1 (segregation) *, PP3 (in silico)	Likely disease-causing without definitive genetic proof	Used for working diagnosis and family testing of clearly symptomatic or affected relatives to confirm family segregation. Strictly NO predictive screening of asymptomatic relatives or children.
Class IV	Likely Pathogenic	>90% probability of pathogenicity; supported by combinations of PM2 (rare), PP3 (in silico), PS3 (functional), PP1 (segregation), PP4 (phenotype)	Likely disease-causing	Used for clinical decisions and standard medical management.
Class V	Pathogenic	Definitive evidence of pathogenicity (e.g., PS1–PS4 with supporting PM and PP criteria)	Disease-causing	Confirms diagnosis; utilized for cascade family testing and clinical screening.

* Note: Critically short telomere length and specific phenotype match (PP4) are clinical tools that heavily guide the utility of this sub-class in practice. Criterial markers like PM2 and PP1 must be interpreted with caution due to the incomplete penetrance and variable expressivity inherent to familial ILD.

## Data Availability

No new data were created or analyzed in this study.
